# USING AN OLDER ADULT MENTORING PROGRAM TO INCREASE INTEREST IN GERIATRICS AMONG MEDICAL STUDENTS

**DOI:** 10.1093/geroni/igae098.3922

**Published:** 2024-12-31

**Authors:** Shannon Arnette, Madeline McIntyre

**Affiliations:** Virginia Commonwealth University, Richmond, Virginia, United States; VCU, Richmond, Virginia, United States

## Abstract

Older adults are the largest consumers of healthcare, yet there is a projected shortage of geriatricians in the future. The Older Adult Mentoring Program at a large, urban-serving, mid-Atlantic university aims to educate first-year medical students by increasing their awareness of internal and external ageism within healthcare through three semi-structured interviews conducted by the students with their mentors over two semesters, allowing them to interact with community-dwelling older adults and gain a person-centered perspective of aging. We conducted a quantitative study to determine the program’s impact on the students’ openness to working in geriatrics as a specialty. In April 2024, at the end of the program, we asked the students several questions, such as how they think about their healthcare goals as they age, their comfort level working with older adults, and their openness to pursuing geriatrics. Our findings suggest that the program positively impacted students’ attitudes to working with older adults. Over 75% of students agreed that the program changed how they think about their healthcare needs and/or goals as they age. Approximately 77% of students agreed that they feel more confident working with older adults. Over 60% of students either somewhat agreed or agreed that they are more open to pursuing geriatrics as a specialty, and over 90% were more open to working with older adults regardless of their future specialty. This shows how meaningful a program like this can be in changing students’ attitudes towards older adults and geriatrics.

